# A Novel Approach for Transvenous Embolization of Dural Arteriovenous Fistula Using a Balloon and a Coil as Walls: Case Presentation

**DOI:** 10.1155/2022/5164452

**Published:** 2022-02-25

**Authors:** Kenji Fukutome, Shuta Aketa, Tsukasa Nakajima, Hiromichi Hayami, Hiromitsu Sasaki, Ryuta Matsuoka, Rinsei Tei, Yasushi Shin, Yasushi Motoyama

**Affiliations:** Department of Neurosurgery, Osaka Police Hospital, Osaka, Japan

## Abstract

**Background:**

Transvenous embolization (TVE) for dural arteriovenous fistula (DAVF) is difficult depending on an accessible route. Reported herein is a case of transvenous embolization using a balloon and a coil as “walls.” *Case Description*. A 56-year-old male patient presented with a 1-month history of mild motor aphasia. The magnetic resonance imaging showed a hemorrhagic lesion in his left temporal lobe, and the cerebral angiography showed a DAVF, with parasinus shunt points near the torcula and the left transverse sinus. Access to the shunt point was very difficult; however, TVE was performed using a balloon as a wall. Furthermore, all lesion embolization was possible using a coil as a wall.

**Conclusions:**

Using a balloon or coil as a wall during a TVE is useful.

## 1. Introduction

Transvenous embolization (TVE) is a common treatment for dural arteriovenous fistula (DAVF), but its approach is difficult depending on the access route. Reported herein is a case of DAVF with a shunt point approach, and TVE was performed using a balloon and a coil as “walls.”

## 2. Case Description

**Figure 1:**
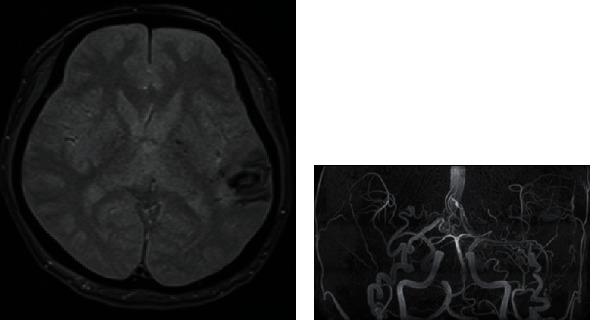
(a) T2 star-weighted magnetic resonance imaging revealed a hemorrhagic lesion in his left temporal lobe. (b) Magnetic resonance angiography revealed abnormal sinus signals.

A 56-year-old male patient, without medical history, presented with a 1-month history of mild motor aphasia. He was able to speak without any problems but sometimes stuck in words. The T2 star-weighted magnetic resonance imaging (MRI) and magnetic resonance angiography of the brain revealed a hemorrhagic lesion in the left temporal lobe (Figure 1(a)) and sinus signal suspected of DAVF (Figure 1(b)).

**Figure 2:**
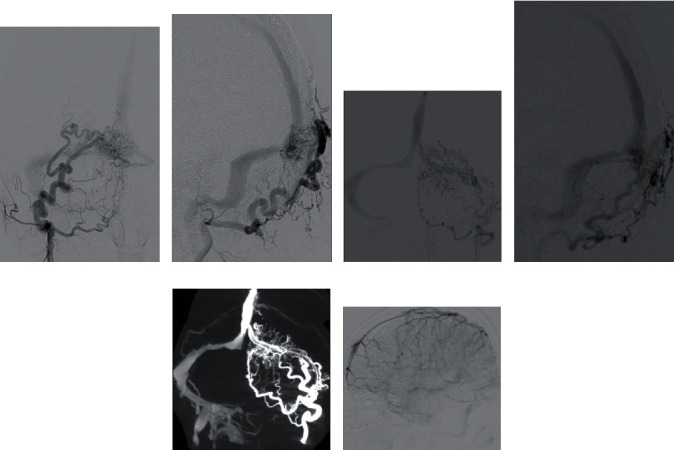
(a–e) Bilateral occipital artery angiography revealed a dural arteriovenous fistula, wherein feeding arteries were bilateral occipital artery, shunt points were a parasinus near the torcula and the left transverse sinus (TS), drainer was a part of the left transverse sinus, left TS was partially occluded, and sinus flow of the superior sagittal sinus (SSS) was anterograde and retrograde. (a) A-P view of right OAG, (b) lateral view of right OAG, (c) A-P view of left OAG, and (d) lateral view of left OAG) (f) Venous phase of left internal carotid artery angiography showed venous congestion without venous cortical reflux. OAG: occipital artery angiography.

The cerebral angiography revealed a DAVF, with feeding arteries from the bilateral occipital artery, parasinus shunt points near the torcula and left transverse sinus (TS), drainer of the left TS, occluded partially left TS, and anterograde and retrograde sinus flow of the superior sagittal sinus (SSS) (Figures 2(a)–2(e)). There was no cortical venous reflux; however, there was venous congestion on a venous phase of left internal carotid artery angiography (Figure 2(f)). The Cognard classification was type IIa, and the Borden classification was type I. TVE was planned to prevent rebleeding. However, the angle from SSS to the drainer was steep; thus, changing to transarterial embolization (TAE) was planned if the approach to the drainer is difficult.

**Figure 3:**
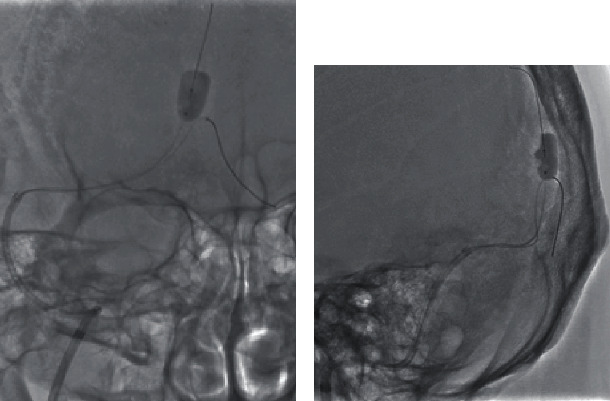
Overinflated SHORYU HR of 7 mm × 7 mm placed in the SSS slightly cranial of the entrance of the left transverse sinus allowed the SL-10 to enter into the left transverse sinus with the Traxcess ((a) A–P view and (b) lateral view).

**Figure 4:**
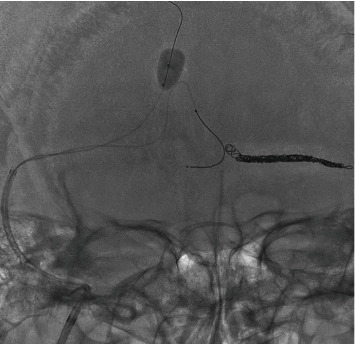
Previously embolized parasinus near left transverse sinus became a wall. It enabled the Traxcess and SL-10 to easily enter into the parasinus near the torcula.

**Figure 5:**
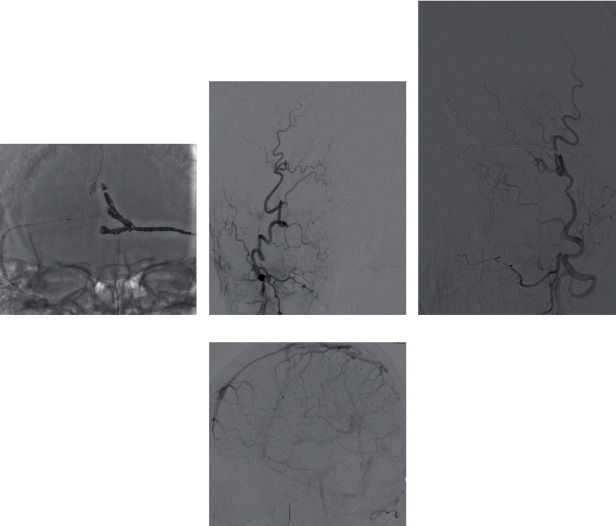
(a–c) The shunt completely disappeared after the embolization from the shunt point near the torcula to the exit of the superior sagittal sinus. ((a) A–P view of X-ray, (b) A-P view of right OAG, and (c) A–P view of left OAG) (d) Venous phase of left internal carotid artery angiography showed a little improvement of venous congestion. OAG: occipital artery angiography.

Under local anesthesia, the right femoral artery was punctured, and a 90 cm 4-French (Fr) FUBUKI (ASAHI INTECC, Aichi, Japan) was introduced as a guiding sheath and advanced into the right external carotid artery for intraoperative angiography and TAE. The right femoral vein was also punctured, and a 25 cm 8-Fr sheath (Terumo, Tokyo, Japan) was introduced. A 90 cm 8-Fr Launcher (Medtronic, Minneapolis, MN) was advanced into the right jugular vein through the sheath. A 113 cm 6-Fr Cerulean DD6 (Medikit, Tokyo, Japan) was used as a distal access catheter, and an Excelsior SL-10 (Striker, Kalamazoo, MI) was made to enter the left TS with a Traxcess (Terumo); however, it was impossible since the Traxcess bounced to the cranial side of the SSS. Therefore, a SHOURYU HR 7 mm × 7 mm (Kaneka Medics, Osaka, Japan) was guided into the SSS, slightly cranial to the entrance of the left TS and used as a wall. The balloon was small as a wall in the sinus at normal inflation; thus, the balloon was overinflated (Figure 3). Nevertheless, the SSS was not completely occluded, but the balloon functioned well as a wall, which allowed the SL-10 to enter into the left TS with the Traxcess. Although the SSS was not completely occluded, just in case, the inflation was done intermittently every 5 minutes. The shunt point was the parasinus near the torcula and the left TS, but the approach to the parasinus near the torcula was difficult due to the steep branching; thus, the parasinus near the left TS was first approached. The SL-10 was allowed to enter the parasinus near the TS, and the arterial side beyond the shunt point is easily reached. First, the parasinus near the left TS was embolized using a total 62 cm coil to return from this part. Therefore, a wall was created, and the Traxcess and Sl-10 were inverted and easily approached the parasinus near the torcula using that wall (Figure 4). The shunt completely disappeared after the embolization using a total of 149 cm of coil from the shunt point to the exit of the SSS (Figures 5(a)–5(c)), and venous congestion has improved a little compared to before the treatment (Figure 5(d)). Finally, each sheath was removed, hemostasis was performed by manual compression, and the operation was completed. No complications were observed after the operation, and his aphasia has almost disappeared.

## 3. Discussion

TVE is an established treatment for DAVF, and the DAVF occlusion rate by TVE was reported as 71%–90% [1–4]. Many cases of TVE for DAVF, such as cavernous sinus and isolated sinus, requiring a difficult approach were reported so far [5, 6]. There are some reports on catheterization with balloon assist in TAE and aneurysm embolization [7–10]. However, no concrete studies were reported about successful approaches using a balloon as a “wall” for TVE. In our case, overinflated SHOURYU HR of 7 mm × 7 mm was used as a wall due to the unavailability of a larger balloon, such as Copernic RC 8 mm × 80 mm (Balt, Montmorency, France) in Japan. If one balloon is unable to create a large enough wall, placing the two in parallel is possible. The strategy was different from ours, and the balloon was not used to assist the approach; however, Yabuzaki et al. reported a case of DAVF treated using two balloons in the sinus [11]. The duration of complete occlusion of the sinus, including SSS, is unknown, but even with incomplete occlusion as in our case, the balloon can function well as a wall. Therefore, it is not necessary to completely occlude the sinus. There is a report of using a coil mass as an assist in TVE to induce the microcatheter to turn back to the shunting point [12]; however, the use of a coil as a wall is controversial. There is no problem if the route is sufficiently packed with a sufficient amount of coil as in our case, but if the amount of coil is not sufficient, the coil is pushed in, making it dangerous.

## 4. Conclusions

Approaching the shunt point during TVE is difficult; thus, a balloon or a coil as a wall is useful.

## Data Availability

The data used to support the findings of this study are available from the corresponding author upon request.
